# Growth of preterm very low birth weight infants discharged with weight of less than 1500grams

**DOI:** 10.1186/s12887-021-02612-4

**Published:** 2021-03-25

**Authors:** Yaser Abdallah, Flavia Namiiro, Jolly Nankunda, Jamiru Mugalu, Yvonne Vaucher

**Affiliations:** 1 Department of Paediatrics and Child Health, Makerere University College of Health Sciences, P.O Box 7072, Kampala, Uganda; 2 Department of Paediatrics and Child Health, Mulago National Referral hospital, Kampala, Uganda; 3 Department of Pediatrics, Division of Neonatal/Perinatal Medicine, School of Medicine, University of California at San Diego, San Diego, USA

## Abstract

**Abstract:**

Early discharge of preterm very low birth weight (VLBW) infants is at times inevitable in low resource settings. The implication of such practice on the growth of this high-risk population is not known. We conducted a retrospective chart review to describe the growth of preterm VLBW infants discharged with a weight of less than 1500 g.

**Objectives:**

To describe the growth of discharged preterm VLBW infants over the first 12 weeks.

**Method:**

Between June 2013 and January 2014; 164 discharged preterm VLBW infants were followed up for 3 months. Among the survivors (132), we identified 111 infant records for this study. Relevant data was entered in STATA for analysis. Growth percentiles were determined at approximately 4 weeks, 8 weeks, and 12 weeks post-discharge using the intergrowth 21st growth charts. Growth velocities were computed using the 2-point average weight model. Regression analysis was used to identify factors associated with growth failure. Growth failure was defined as occipital frontal circumference (OFC), weight, and length < 10th centile by 12 weeks post-discharge. *P*-value of < 0.05 was considered significant at a 95% confidence interval.

**Results:**

Among the study infants the median gestational age and weight at birth were 32 weeks (range 28-35 weeks) and 1250 g(range 850-1500 g) respectively; 60/111(54%) were Small for Gestational Age (SGA). The median discharge postmenstrual age (PMA) was 34 weeks (range 30-38 weeks) and weight was 1140 g (range 830-1490 g). The majority 88.2% had not recovered birth weight at discharge of whom 59.1% recovered by 2 weeks and 40.9% recovered between 2 and 4 weeks after discharge. By 12 weeks post-discharge the median PMA and weight were 46 weeks (range 37-51 weeks),and 3110 g (range 1750-5000 g) respectively, 38.7% of the infants had growth failure and 36.9% had OFC <3rd centile. Growth velocity < 15 g/kg/d in the first 4 weeks (OR 3.8, p 0.010) and subsequent 4 weeks (OR 2.5, p 0.049) post-discharge were independently associated with growth failure.

**Conclusion:**

Slow birth weight recovery was observed and growth failure was prevalent by 12 weeks post-discharge with more than a third having severe microcephaly. Poor post-discharge growth velocity was associated with subsequent growth failure.

**Recommendations:**

Growth velocity monitoring among preterm VLBW infants should be emphasized. The implication and interventions of this early growth failure needs to be explored.

## Background

It was estimated in 2015 that approximately 20.5million babies were born with low birth weight and the majority 91% occurred in low and middle-income countries [1]. Low birth weight babies account for 80% of global neonatal deaths [2, 3] of these two-thirds are preterm [1–3]. Surviving preterm and low birth weight babies have been observed to be at risk for morbidities ranging from stunting, ill-health, and adult-onset chronic conditions like cardiovascular diseases among others [4–7]. Low birth weight (LBW) has been associated with the loss of human capital through an increase in high school dropout rate, lowering labor force participation, and early aging [8].

One of the concerns among surviving preterm infants is their growth and its health implications. Various studies have demonstrated that poor growth among preterm very low birth weight infants (VLBW) is associated with poor neurodevelopmental outcomes hence emphasizing the need for promotion and maintaining close to normal early growth among these infants [7, 9, 10]. Nutrition among these infants aims to mimic normal fetal growth to promote development [11].

In the developed countries preterm and VLBW infants are commenced on parenteral nutrition early in life and once on full feeds, their nutritional requirements are adjusted to meet the European Society Paediatric Gastroenterology Hepatology and Nutrition (ESPHAGAN) recommendations [11]. In Low and Middle-income Countries (LMIC) the WHO (world Health Organization) guideline on the feeding of LBW infants is followed, this guideline follows weight gain to decide the next appropriate intervention [12].

At the Mulago National referral hospital, VLBW infants do not routinely receive parenteral nutrition and are discharged home early due to lack of space in the hospital. Parents are educated on how to gavage and breastfeed their infants. Feeding and weight gain are reviewed weekly. Escalation of feeding to fortified breast milk does not happen even among poorly growing LBW infants due to cost and availability.

A previous follow-up of LBW infants at Mulago hospital kangaroo clinic observed the majority of babies not recovering birth weight by 21 days of life [13]. We describe here the growth among a cohort of VLBW infants discharged with a weight of < 1500 g from the Mulago Special Care Baby Unit (SCBU) over the first 12 weeks post-discharge to guide post-discharge follow-up for such infants.

## Methods

### Setting

This study was conducted at the Mulago Hospital SCBU. The SCBU is a level II neonatal unit that admits approximately 4500 babies annually of whom 20% are VLBW. Mulago hospital is a National Referral Hospital for Uganda and a teaching hospital for Makerere University.

In terms of nutrition; neonates in the unit are commenced on intravenous fluids containing dextrose as breast milk feeds are introduced slowly. Total fluid requirements are computed daily and are provided as feeds together with supplemental intravenous fluids. Electrolytes are added to intravenous fluids from day 3 of life. Neither intravenous lipids nor amino acids are given routinely. When a neonate is tolerating full feeds (150 ml/kg/day) then intravenous fluids are discontinued.

Once preterm and low birth weight babies are stable (i.e., maintaining stable body temperature while in kangaroo care, not needing supplemental oxygen and tolerating feeds), they are discharged home on oral aminophylline, multivitamin drops (Grovit), iron syrup with follow-up once weekly in the Kangaroo clinic located in the SCBU. Most preterm infants are discharged while still tube feeding.

In the Kangaroo clinic anthropometry is done by a trained nurse. Length is measured using an infant length board calibrated to +/− 0.5 cm accuracy, naked weight by a digital weighing scale calibrated to +/− 10 g accuracy, and OFC by a none stretchable tape measure. All measurements are performed according to WHO guidelines [14]. Babies are assessed by a paediatrician or a resident in paediatrics. On average the clinic sees 30–40 babies per day.

Babies found with any complication may be readmitted for care while for stable ones; parents are advised on continued care and are given their next appointment. Feed volumes are readjusted (maximum feeding volume is 220 ml/kg/day), and nasogastric tubes are replaced. Breast milk is never fortified.

Cup feeding and breastfeeding are introduced once the infant shows sucking gestures. If cup feed volume exceeds half daily volume then the nasogastric tube is removed and once sucking on the breast is perceived strong and sustained then full breastfeeding is commenced.

### Study population

The infants included in this cohort were VLBW (birth weight < 1500 g), had been discharged home with a weight of < 1500 g, and did not have comorbidities.

### Procedure

Data sheets of all infants enrolled in the previous study on mortality among VLBW infants following hospital discharge were reviewed [15]. Infants who did not survive at 12 weeks post-discharge were excluded from the study. Relevant study variables included: sex, gestational age, hospitalizations, weight, length, and head circumference at birth, discharge, 4 weeks, 8 weeks, and 12 weeks post-discharge. Other variables were maternal age, parity, mode of feeding, kangaroo hours, Human Immunodeficiency Virus (HIV) status. Only complete data sheets were included in the analysis. Growth failure was defined as having weight, OFC, and length below 10th centile at 12 weeks post-discharge using the 21st intergrowth chart [16].

#### Data management and analysis

Data were entered into the computer using the Microsoft Excel program and subsequently exported into the STATA software package for analysis. The intergrowth online tool was used to compute weight, OFC, and length centile at the different postmenstrual ages.

Growth velocity was computed as

$$ \mathrm{GV}=\left[\frac{\left(\mathrm{Wte}\right.\left(\mathrm{g}\right)\hbox{-} \mathrm{Wts}\left(\mathrm{g}\right)}{\mathrm{P}}\right]\div \frac{\mathrm{Wte}\left(\mathrm{kg}\right)+\mathrm{Wts}\left(\mathrm{kg}\right)}{2} $$

GV = Growth Velocity.

Wte = weight at end of assessment.

Wts = weight at start of assessment.

P = Period of assessment in days (age at Wte less age at Wts).

The Pearson Chi-square test was performed to compare baseline characteristics between included and excluded study participants.

Using baseline variables logistic regression was run to identify factors independently associated with growth failure. *P* values of < 0.05 were considered significant and the confidence interval of 95% was used. The results were summarized in tables.

## Results

Of the 132 preterm VLBW (< 1500 g), infants born between June 2013 and January 2014 who were known to be alive at 12 weeks post-discharge, we identified 111 infants (53 males and 58 females) with complete records. The median gestational age and weight at birth were 32.5 weeks (range 28-35 weeks) and 1250 g (range (850-1500 g) respectively. Sixty were SGA of whom 45/111 (40.5%) and 15/111(13.5%) were symmetrically and asymmetrically SGA respectively at birth. Baseline cohort characteristics are shown in Table 1.

**Table 1:** Comparison of Baseline characteristics and outcomes of study participants

Variables Baseline characteristics	Included participants N: 111 (%)	Excluded participants N: 21(%)	p-value
**Sex**			
Male	53 (47.7%)	2 (9.5%)	
Female	58 (52.2%)	19 (90.5%)	0.008
**Gestational Age at discharge**			
≤ 34 weeks	60 (54.0%)	8 (38.1%)	
> 34 weeks	51(46.0%)	13(61.9%)	0.189
**Weight at discharge**			
≤ 1200 g	73 (65.7%)	15 (71.5%)	
> 1200 g	38 (34.3%)	6 (28.5%)	0.614
**OFC at birth**			
≥ 10th centile	59 (53.1%)	10 (47.6%)	
< 10th centile	52 (46.8%)	11(52.4%)	0.642
**Small for Gestational Age**			
Yes	60 (54.0%)	14 (66.6%)	
No	51 (46.0%)	7 (33.4%)	0.536
**HIV exposure**			
Yes	18 (16.2%)	5 (23.8%)	0.403
No	93 (83.8%)	16 (76.2%)	
**Twins**			
Yes	16 (14.4%)	1(4.8%)	0.251
No	95 (85.6%)	20 (95.2%)	
**Maternal parity**			
< 2	33 (29.7%)	8 (38.1%)	
≥ 2	78 (70.3%)	13 (61.9%)	0.450
**Maternal education**			
None/primary	43 (38,7%)	6 (28.6%)	
Secondary/tertiary	68 (61.3%)	15 (71.4%)	
**Maternal income**			
< 100,000 Ugandan Shillings.	76 (68.5%)	6 (28.6%)	
≥100,000 Ugandan Shillings.	35 (31.5%)	15 (71.4%)	
**Outcome variables**			
**1st-month KMC hrs**			
< 12 h	83 (74.7%)	7 (33.3%)	
≥ 12 h	28 (25.3%)	6 (28.6%)	
Unknown	0 (0.0%)	8 (38.0%)	
**Birth weight recovery 2 weeks post-discharge**			
Yes	71(63.9%)	8 (38.1%)	
No	40 (36.1%)	8 (38.1%)	
Unknown	0 (0.0%)	5(23.8%)	
**KMC support**			
Yes	24 (21.6%)	5 (23.8%)	
No	87 (78.4%)	16 (76.2%)	
**Weight 12 weeks after discharge**			
≥ 10th centile	28 (25.1%)	5 (23.8%)	
< 10th centile	83 (74.9%)	8 (38.1%)	
Unknown	0 (0.0%)	8 (38.1%)	
**OFC 12 weeks after discharge**			
≥ 10th centile	68 (61.3%)	6 (28.6%)	
< 10th centile	43 (38.7%) ª	4 (19.0%)	
Unknown	0 (0.0%)	11 (52.4%)	

ª: 41 of the 43 had OFC <3rd centile

Although most of infants excluded from analysis were female 19/21 (p 0.008), the baseline characteristics among included and excluded infants were not statistically significant Table 1.

The average duration of hospital stay was 12 days (range 4-41 days). The median discharge PMA and weight were 34 weeks (range 30-38 weeks) and 1140 g (range 830-1490 g). Birth weight was recovered in only 13(11.7%) infants at discharge, in whom the average time to birth weight recovery was at 12.6 days (range7-21 day).

The majority of the infants at discharge were still tube feeding 100/111. By 12 weeks after discharge 107/111 infants were exclusively breastfeeding directly from the breast and 4 were taking formula feeds.

Of the infants who had not recovered birth weight at discharge 98 (88.2%); 70 (71.4%) weighed ≤1200 g at discharge. Among these infants 58(59.1%) recovered birth weight by 2 weeks after discharge. Among those who recovered birth weight between 2 and 4 weeks after discharge 40(40.8%) the majority 34/40 were those discharged with weight of ≤1200 g.

In the first 4 weeks post-discharge, 28mothers were practicing Kangaroo Mother Care (KMC) for 12-20 h per day; by 8 weeks post-discharge; 9 mothers were doing so (Table 2).

**Table 2:** Post-discharge gestational ages, weight, and KMC hours

Weeks Post-discharge	Median Post-menstrual age (range)	Number of infants with Gestational age < 35 weeks	Median weight (range) grams	Number of infants with weight < 2500 g	Number of infants Receiving KMC		
					> 20 h	12-20 h	< 12 h
**4 weeks**	39 (34–41)	9	1630 (1030–2800)	108	0	28	83
**8 weeks**	42 (36–46)	1	2477 (1280–4220)	62	0	9	102
**12 weeks**	45 (37–51)	0	3293 (1750–5000)	10	0	2	107

Between discharge and 4 weeks post-discharge 8 infants were hospitalized for possible sepsis, 3 of them subsequently had growth failure; between 4 and 8 weeks post-discharge 1 infant was hospitalized for anemia and between 8 and 12 weeks 2 infants were hospitalized for pneumonia.

By12 weeks post-discharge the median PMA and weight were 46 weeks (range 37-51 weeks) and 3110 g (range 1750-5000 g) respectively. The majority of the infants had weight 73.8% and length 72.6% below 10th centile. Growth failure was evident in 43/111 (38.7%) infants. Forty-one (36.9%) infants had OFC < 3rd centile (Table 1).

**Fig. 1:**
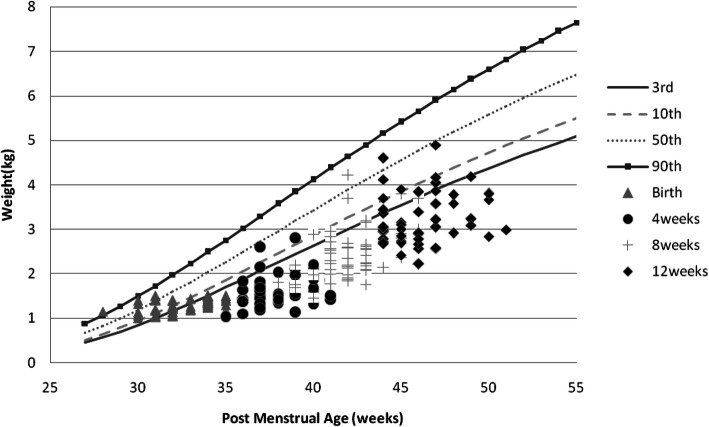
Post-discharge weight changes of Preterm/VLBW boys

**Fig. 2:**
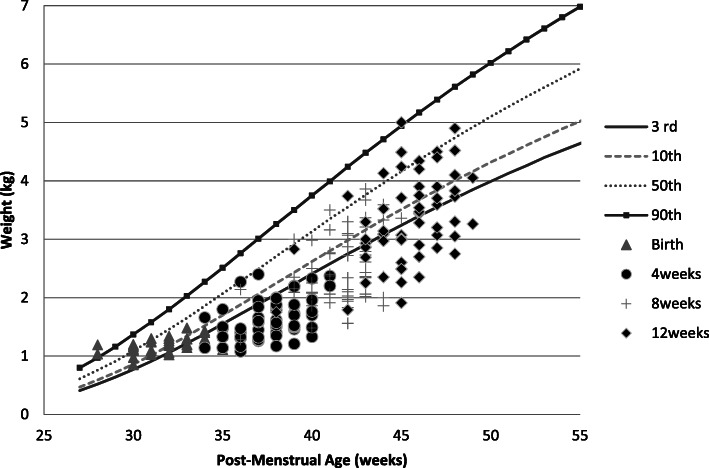
Post-discharge weight changes of Preterm/VLBW girls

**Fig. 3:**
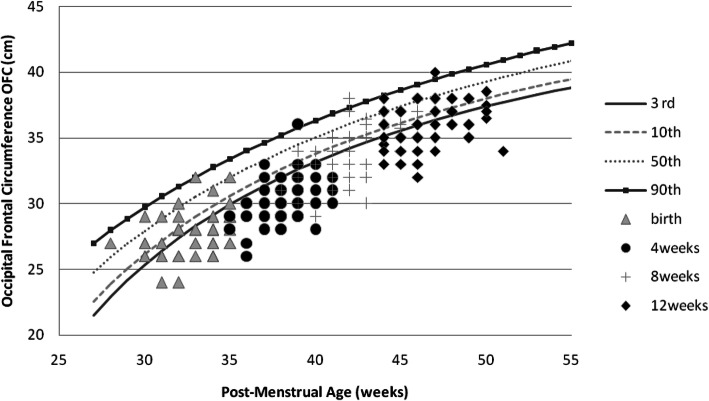
Post-discharge head growth of Preterm/VLBW boys

**Fig. 4:**
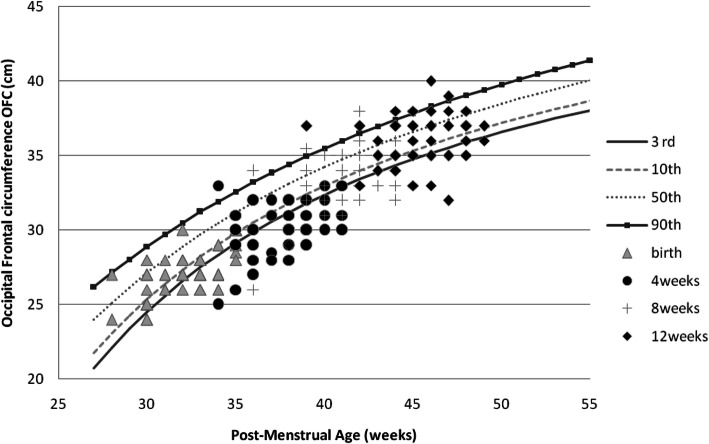
Post-discharge head growth of Preterm/VLBW girls

**Fig. 5:**
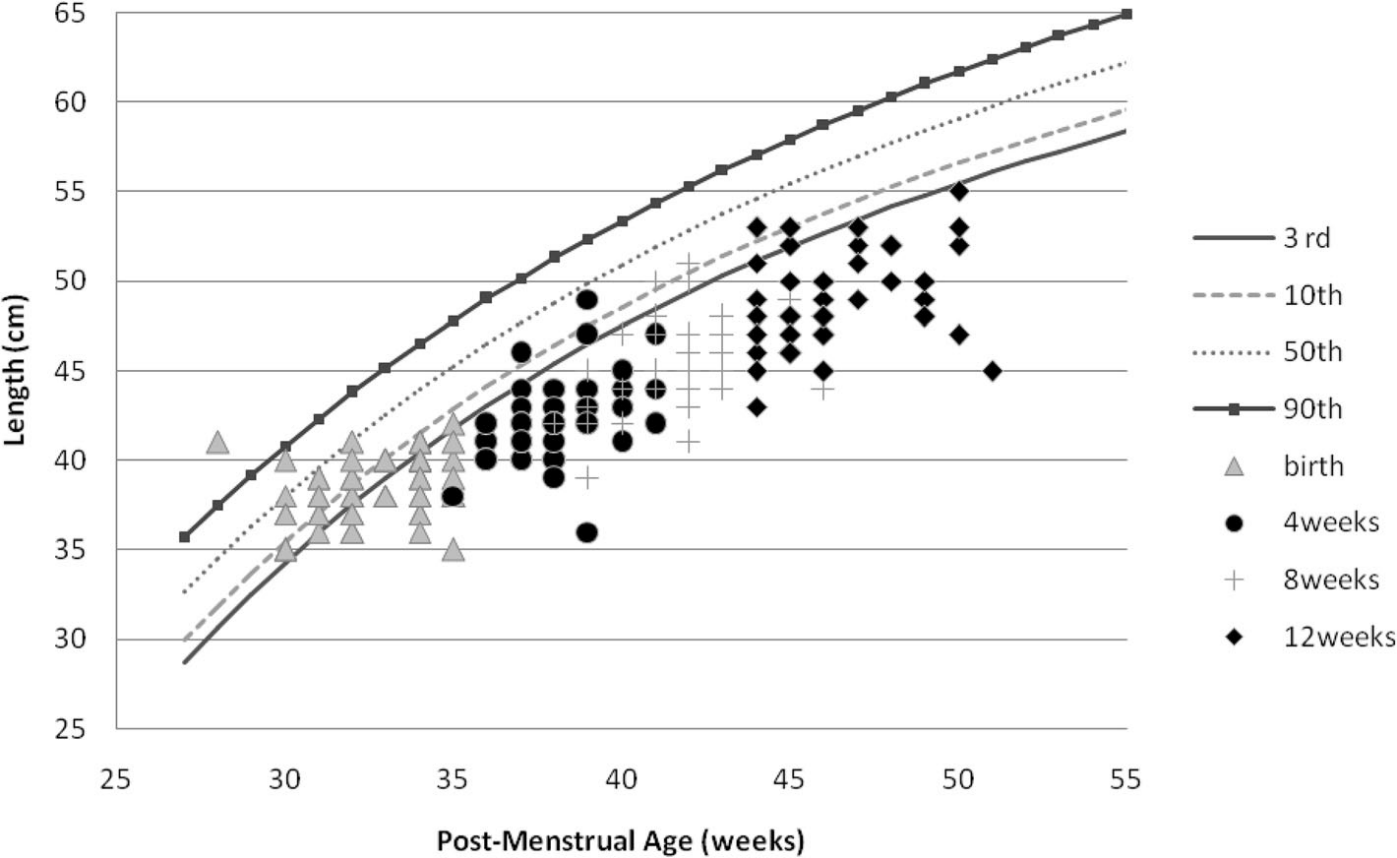
Post-discharge length changes of Preterm/VLBW boys

**Fig. 6:**
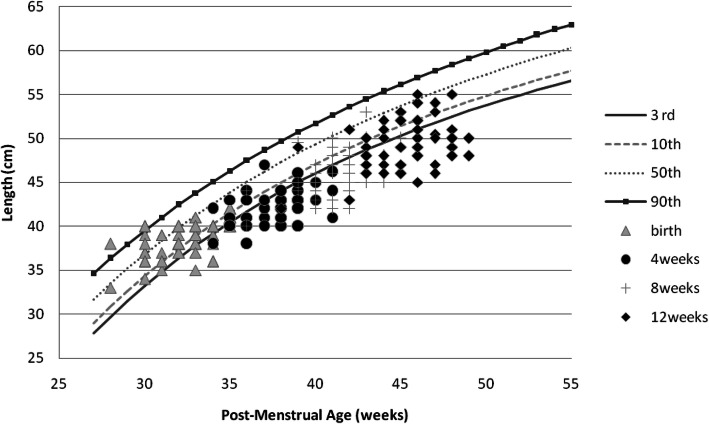
Post-discharge length changes of Preterm/VLBW girls

Girls had an overall better weight, length, and OFC recovery than boys (Figs. 1, 2, 3, 4, 5 and 6 and Table 3).

**Table 3:** Comparison of growth between girls and boys

Anthropometry	Boys N: 53(%)	Girls N: 58 (%)	Total N: 111 (%)
**Birth weight**			
≥ 10th centile	23 (43.4%)	28(48.2%)	51(46.0%)
< 10th centile	30(56.6%)	30(51.8%)	60 (54.0%)
**Weight 12 weeks post discharge**			
≥ 10th centile	9(17.0%)	19(32.8%)	28(25.2%)
< 10th centile	44(83.0%)	39(67.2%)	83(74.8%)^b^
**OFC birth**			
≥ 10th centile	21(39.6%)	38(65.5%)	59(53.1%)
< 10th centile	32(60.4%)	20(34.5%)	52(46.9%)
**OFC 12 weeks post discharge**			
≥ 10th centile	28(52.8%)	40(69.0%)	68(61.3%)
< 10th centile	25(47.2%)	18(31.0%)	43(38.7%)^c^
**Length birth**			
≥ 10th centile	24(45.3%)	39(67.2%)	63(56.7%)
< 10th centile	29(54.7%)	19(32.8%)	48(43.3%)
**Length 12 weeks post discharge**			
≥ 10th centile	7(13.2%)	23(39.6%)	30(27.0%)
< 10th centile	46(86.8%)	35(60.4%)	81(73.0%)
**Symmetric SGA at birth**^a^			
**Yes**	25(47.1%)	20(34.5%)	45(40.5%)
**No**	28(52.8%)	38(65.5%)	66(59.5%)

^a^: Symmetric SGA = Weight, FC and Length < 10th centile for gestational age

^b^: Of these 83; 48 had weight < 10thcentile at birth

^c^: Of these 43; 26 had OFC <10th centile at birth

## Discussion

From this analysis, 38.7% of the preterm VLBW infants had growth failure with weight, length and head circumference below the 10th percentile for PMA by 12 weeks post-discharge. Growth failure was more frequent among SGA infants 28/60 (46.6%) compared to 15/51(29.4%) of non-SGA infants although this difference was statistically not significant (p 0.06) (Table 4).

**Table 4:** Factors associated with growth failure

Variables	No Growth failure N: 68(%)	Growth failure N: 43(%)	COR	p-value	AOR	p-value
**Sex**						
Male	28(41.2%)	25(58.1%)	1.98 (0.91–4.30)	0.081	1.23 (0.50–3.00)	0.644
Female	40 (58.8%)	18(41.9%)				
**Gestational Age at discharge**						
≤ 34 weeks	39(57.3%)	21(48.8%)	0.71 (0.33–1.53)	0.38		
> 34 weeks	29 (42.7%)	22(51.2%)				
**Weight at discharge**						
≤ 1200 g	43(63.2%)	30(69.8%)	1.34 (0.96–4.62)	0.48		
> 1200 g	25(36.8%)	13(30.2%)				
**Small for Gestational Age**						
Yes	32(47.0%)	28(65.1%)	2.10 (0.95–4.61)	0.064	2.30 (0.94–5.62)	0.06
No	36(53.0%)	15(34.9%)				
**HIV exposure**						
Yes	10(14.7%)	8(18.6%)	1.33 (0.47–3.67)	0.588		
No	58(85.3%)	35(81.4%)				
**Twins**						
Yes	10(14.7%)	6(14.0%)	0.94 (0.31–2.80)	0.912		
No	58(85.3%)	37(86.0%)				
**1st-month KMC hrs**						
< 12 h	51(75.0%)	32(74.4%)	0.97 (0.40–2.33)	0.94		
≥ 12 h	17(25.0%)	11(25.6%)				
**Birth weight recovery 2 weeks post-discharge**						
Yes	50(73.5%)	21(48.8%)	2,91 (1.30–6.50)	**0.009**	2.26 (0.92–5.54)	0.072
No	18(26.5%)	22(51.2%)				
**Growth velocity 1 (discharge to 4 weeks later)**						
< 15 g/kg/day	37(54.4%)	36(83.7%)	4.31 (1.68–11.0)	**0.002**	**3.89 (1.38–10.9)**	**0.010**
≥ 15 g/kg/day	31(45.6%)	7(16.3%)				
**Growth velocity 2 (4to 8 weeks after discharge)**						
< 15 g/kg/day	35(51.5%)	32(74.4%)	2.74 (1.19–6.32)	**0.018**	**2.50 (1.01–6.27)**	**0.049**
≥ 15 g/kg/day	33(48.5%)	11(25.6%)				
**Growth velocity 3** **(8 to12weeks after discharge)**						
< 15 g/kg/day	61(89.7%)	37(86.0%)	0.71 (0.22–2.27)	0.561		
≥ 15 g/kg/day	7(10.3%)	6(14.0%)				
**Maternal parity**						
< 2	21(31.0%)	12(27.9%)	0.866 (0.37–2.01)	0.738		
≥ 2	47(69.0%)	31(72.1%)				
**Maternal education**						
None/primary	29(42.6%)	14(32.5%)	0.64 (0.29–1.44)	0.289		
Secondary/tertiary	39(57.4%)	29(67.5%)				
**Maternal income**						
< 100,000 Ugandan Shs.	50(73.5%)	26(60.4%)	0.55 (0.24–1.24)	0.151	0.40 (0.155–1.05)	0.063
≥ 100,000 Ugandan Shs.	18(26.5%)	17(39.6%)				
**KMC support**						
Yes	15(22.0%)	9(20.9%)	1.07 (0.42–2.72)	0.88		
No	53(78.0%)	34(79.1%)				

Only 13 (11.7%) of the study infants had recovered birth weight at the time of hospital discharge. Of these 8 were appropriately grown for gestational age and 5 were SGA at birth. This very slow birth weight recovery is comparable to findings from a study conducted in the same facility among none SGA low birth weight infants [13], but slower than findings from other studies [17–19]. This observation can be explained by the difference in practice between our facility and others. In our facility, VLBW infants do not routinely receive total parenteral nutrition as enteral feeds are introduced.

Of the 98/111(88.2%) discharged home before birth weight recovery; 58(59.1%) recovered birth weight by 2 weeks after discharge and 40(40.9%) recovered 2-4 weeks after discharge. Whereas discharge weight of ≤1200 g did not seem to be significantly associated with growth failure (OR 1.34 p 0.48) (Table 4), the majority of infants 34/40(85%) who had not recovered birth weight by 2 weeks post-discharge were those discharged with a weight of ≤1200 g. Infants discharged with weight ≤ 1200 g are likely to be more immature and more susceptible to environmental stressors such as hypothermia accounting for slower growth among this weight category.

By 4 weeks post-discharge 28/111 (25.2%) infants were reportedly receiving KMC for 12-20 h/day (Table 2). Despite kangaroo mother care being associated with improved growth beyond 40 weeks postmenstrual age [20];our study participants were not receiving continuous KMC as recommended [21, 22] this exposed them to increased caloric needs for maintaining body temperature possibly at the expense of growth. It is important that continuous KMC is emphasized and supported in the community in low resource settings.

Growth velocity < 15 g/kg/d in the first 4 weeks (OR 3.8, p 0.010) and subsequent 4 weeks (OR 2.5, p 0.049) post-discharge were independently associated with growth failure (Table 4). Early post-natal growth has been positively associated with neurodevelopment outcomes among preterm infants [7, 10]. ESPHGAN recommends a growth velocity of 17-20 g/kg/d to prevent preterm infants from dropping off their centile lines [23] and a growth velocity of 16.2+/− 2.4 g/kg/d has been shown to be achievable for VLBW infants on full feeds of 200mls/kg/d once the birth weight has been recovered [18]. Despite being on full feeds; the majority 65.7% of our study infants had growth velocity < 15 g/kg/d (Table 4) between discharge and 4 weeks post-discharge. This finding can be explained by the accrued negative protein balance our study participants encounter since parenteral amino acids were not provided as enteral feeds were introduced during their hospital stay.

The majority of our study infants 59/111 (53.1%) had normal OFC at birth of these 17(28.8%) had OFC below the 10th centile by 12 weeks post-discharge while those who had OFC below 10th centile at birth 52/111(46.9%) by 12 weeks post-discharge 26 (50%) still had OFC <10th centile (Table 3). From Figs. 3 and 4 it is evident that OFC dropped off the centile lines but by 12 weeks post-discharge there seems to be a recovery of OFC compared to weight and length Figs. 1, 2, 5 and 6. Poor early post-natal head growth has been associated with adverse neurodevelopmental outcomes [9], this may imply a higher neurodevelopmental impairment for our study infants.

We noted both boys and girls drifted off their centile lines by 4 weeks post discharge (Figs. 1 and 2), by 8 weeks post discharge girls started recovering back to their appropriate centile line for PMA and by 12 weeks post discharge 32.8% of girls were above the 10th centile compared to 17% of boys (Table 3), similar trend is observed with length (Figs. 5 and 6) and by 12 weeks post discharge 39.6% of the girls had length above the 10^th^ centile compared to 13.2% of boys (Table 3). These observations are contrary to what is expected as per intergrowth 21st charts [16] since boys are expected to attain higher anthropometric measurements compared to girls. This observation may be explained by the fact that more boys (47.1%) were symmetrically growth restricted at birth as compared to the girls (34.5%)(Table 3).

Slow birth weight recovery was observed with growth failure prevalent by 12 weeks post-discharge in the majority of VLBW infants in this study with more than a third having severe microcephaly. Enhanced early nutrition has been shown to ameliorate poor postnatal head growth a practice that LMIC should consider in the care of preterm VLBW infants to avert poor early head growth and improve long term neurodevelopmental outcome.

## Conclusion

Growth velocity monitoring among preterm VLBW infants should be emphasized. The implication and interventions of this early growth failure needs to be explored.

### Limitations

We could not ascertain accurately the volume of feeds the study infants received since some of them were breastfeeding as well as receiving top-up feeds by tube/cup. The duration KMC was as reported by the mothers and subject to recall bias.

Considering a large proportion of our study infants were SGA with 40.5% being symmetrically growth restricted and these infants showing higher tendency to growth failure, other intrinsic factor beyond this study for growth failure among this population cannot be excluded.

We did not analyze for factors associated with growth failure among SGA because of a small sample size and possibility of intrinsic factors for which our study was not design to identify.

### What is known

Preterm very low birth weight infants are at risk for growth failure.

### What this study adds

Early discharge with weight of less than 1500 g may results in slower birth weight recovery and high growth failure.

## Data Availability

The data from this study is available with the corresponding author and can be accessed on request.

## Abbreviations

ESPGHAN: European Society for Pediatric Gastroenterology Hepatology and Nutrition
HIV: Human Immunodeficiency Virus
KMC: Kangaroo Mother Care
LBW: Low Birth Weight
MLIC: Middle and Low Income Countries
OFC: Occipital Frontal Circumference
PMA: Post-Menstrual Age
SCBU: Special Care Baby Unit
SGA: Small for Gestational Age
VLBW: Very Low Birth Weight
WHO: World Health Organization
